# Bowel Preparation and Subsequent Colonoscopy Is Associated with the Risk of Atrial Fibrillation: A Population-Based Case-Crossover Study

**DOI:** 10.3390/jpm12081207

**Published:** 2022-07-25

**Authors:** Yoon Suk Jung, Yongho Jee, Eui Im, Min-ho Kim, Chang Mo Moon

**Affiliations:** 1 Division of Gastroenterology, Department of Internal Medicine, Kangbuk Samsung Hospital, Sungkyunkwan University School of Medicine, Seoul 03181, Korea; ys810.jung@samsung.com; 2 Advanced Biomedical Research Institute, Ewha Womans University Seoul Hospital, Seoul 07804, Korea; jyongho@ewha.ac.kr; 3 Division of Cardiology, Department of Internal Medicine, Yonsei University College of Medicine and Cardiovascular Center, Yongin Severance Hospital, Yongin 16995, Korea; imeui97@yuhs.ac; 4 Informatization Department, Ewha Womans University Seoul Hospital, Seoul 07804, Korea; mino-kim@naver.com; 5 Department of Internal Medicine, College of Medicine, Ewha Womans University, Seoul 07985, Korea; 6 Inflammation-Cancer Microenvironment Research Center, College of Medicine, Ewha Womans University, Seoul 07804, Korea

**Keywords:** bowel preparation, atrial fibrillation, colonoscopy

## Abstract

This study aimed to clarify the association of the risk of atrial fibrillation (AF) with bowel preparation and subsequent colonoscopy through population-based case-crossover analysis. Patients who developed new-onset AF after undergoing colonoscopy following bowel preparation were included. For each patient, one hazard period and four control periods were matched at specified time windows. Among 189,613 patients with AF, 84 patients (mean age: 72.4 years) finally met the inclusion criteria. Most patients used polyethylene glycol (PEG)-based solutions (2 L PEG + ascorbic acid (*n* = 56), 4 L PEG (*n* = 21)) as purgatives and had hypertension (*n* = 75). A significant association of bowel preparation and colonoscopy with AF occurrence was found in all time windows. The proportion of patients with bowel preparation and colonoscopy was higher during the hazard period than during the control periods. In the 1-, 2-, 4-, 8-, and 12-week time windows, the proportions were 11.9% vs. 4.2%, 13.1% vs. 4.8%, 16.7% vs. 6.3%, 28.6% vs. 11.9%, and 29.8% vs. 14.0%, and the odd ratios (ORs) were 3.11, 3.01, 3.00, 2.96, and 2.61, respectively. Bowel preparation and undergoing colonoscopy was associated with the risk of AF and this examination need to be performed with caution especially in elderly patients with hypertension.

## 1. Introduction

Various types of bowel preparation agents have been used to improve the quality of bowel cleansing for colonoscopy [1]. However, these bowel preparation agents can cause adverse events, including electrolyte imbalances and acute renal failure [2,3]. Case reports of rare adverse cardiac events, such as heart failure exacerbation related to bowel preparation, have been published [4,5,6]. Recently, one case report suggested the possible association of purgative use and the development of atrial fibrillation (AF) [7]. The case report presented two patients who developed AF after bowel preparation for colonoscopy. Both patients followed a split bowel preparation protocol with 4 L polyethylene glycol (PEG) and presented AF with a rapid ventricular response before colonoscopy, requiring admission to a telemetry bed [7]. To date, this case series is the only report to suggest a relationship between purgative use and the occurrence of AF. Although several studies have revealed the risk of acute renal failure associated with bowel preparation, no study has investigated AF as a cardiac sequela of bowel preparation.

Some difficulties exist in elucidating the association between purgative use and the risk of AF. The first difficulty is related to study design. To identify drug-related adverse events, observational studies are usually performed. Most observational studies are designed as case-control or cohort studies. However, as these two study designs compare two groups according to exposure to a specific drug, they may have limitations such as selection bias and vulnerability to personal confounders. To overcome these limitations, the case-crossover design, in which each case can serve as its own control, has been developed. A case-crossover design is superior to a case-control or cohort design for the assessment of short-term effects after transient exposures (e.g., risk of AF associated with purgative use), because it eliminates time-invariant confounders between participants and reduces unmeasured confounders [8,9]. The second difficulty in elucidating the relationship between purgative use and the occurrence of AF lies in securing a sufficient number of patients with data on outcomes. Because the incidence of AF is extremely low, hospital-based studies have very limited data for use in assessing the risk of AF after purgative use.

Thereby, we conducted this nationwide population-based case-crossover study using data from a national health insurance database to clarify the association of bowel preparation and subsequent colonoscopy with the risk of AF.

## 2. Materials and Methods

### 2.1. Data Source and Ethical Considerations

Our study was performed using data from the Korean National Health Insurance Service (NHIS) database. Universal medical coverage was achieved in Korea in 1989, and all Koreans are mandatorily enrolled in the NHIS database. Thus, the NHIS database contains data on all claims, including prescribed drugs and procedures, for the entire population of South Korea [10,11,12,13]. Medical claims data submitted between 1 January 2013 and 31 December 2019, were obtained for the current study. 

The study protocol was approved by the Institutional Review Board of Ewha Womans University Mokdong Hospital (approval no. 2021-09-010). The NHIS database is encrypted and does not contain personal identifiers. As this was a retrospective study using only de-identified data, the requirement for informed consent was waived.

### 2.2. Study Design

A case-crossover analysis was performed using cases at previous time points as their own controls, thus keeping the results free from bias caused by time-invariant or personal confounders between participants through within-participant comparisons [14]. A case-crossover study is an appropriate method for evaluating drug safety when the exposure is intermittent, when the effects on risk are immediate and temporary, and when the outcomes are sudden [14,15]. Previous studies have used a case-crossover study design to evaluate the risk of complications related to purgative intake [16,17].

### 2.3. Study Population and Definitions of Variables

Our study population consisted of patients aged ≥50 years who developed new-onset AF after undergoing colonoscopy following bowel preparation using purgatives. Incident AF was defined as the first admission or the first event during at least two different days of hospital visits (outpatient) with a diagnosis of AF (International Classification of Disease 10th revision (ICD-10) code: I48) [18]. 

Colonoscopy was defined as the presence of codes for either colonoscopy, colonoscopic polypectomy, colonoscopic mucosal resection, or colonoscopic submucosal resection. Patients who underwent colonoscopy were considered exposed to purgatives only if the purgative prescription date was within 90 days before the colonoscopy procedure [16,17]. In real clinical practice, some patients are prescribed purgatives but do not undergo colonoscopy for personal reasons. In these situations, it is highly unlikely that these patients actually take the purgatives. All purgatives available in South Korea during the study period were included and analyzed. The types of available purgatives include 4 L PEG, 2 L PEG + ascorbic acid (PEG-A), oral sulfate solution (OSS), sodium picosulfate + magnesium oxide + citric acid (SPMC), sodium picosulfate + PEG + D-sorbitol (SPS), and sodium phosphate (NaP).

The index date was defined as the first date of two or more outpatient visits or hospitalization for AF. To select patients with new-onset AF, those with an AF (I48) or a valvular AF (I050, I052, I342) diagnosis during the preceding 18 months (1 January 2013 to 30 June 2014) were excluded. The 18-month period was selected to ensure that patients with a first incident AF can be included. We used the longest time window option (12 weeks) to calculate the period required before the index date. The required period was 18 months (i.e., 4 × 12-week control periods + 12-week interval + 12-week hazard period). Patients who experienced symptoms related to colon perforation (K63.1, Y60.4, T81.2, K65) within the 14-day period after colonoscopy were excluded. Patients who underwent therapeutic colonoscopic procedures, such as bleeding control (Q7680, Q2062), removal of foreign bodies (Q7670, Q2061), and dilation of colonic stenosis (Q7691, Q7692, Q2065), were also excluded.

### 2.4. Hazard and Control Periods

**Figure 1:**
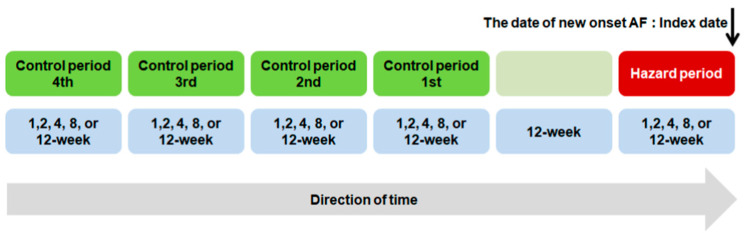
Time sequence of hazard and control periods in this case-crossover study. For each patient with AF, one hazard period and four control periods were paired. Each hazard period was defined as a period of 1, 2, 4, 8, or 12 weeks before the index date. An interval of 12 weeks was selected between the end of the control period and the beginning of the hazard period. For all individual cases, four control periods were also defined as consecutive 1-, 2-, 4-, 8-, and 12-week time windows. AF, atrial fibrillation.

For each patient with AF, one hazard period and four control periods were paired (Figure 1).

We set various time windows (1-, 2-, 4-, 8-, and 12-week periods) to determine the periods for exposure assessment. To elucidate whether the results would fluctuate, each hazard period was defined as a period of 1, 2, 4, 8, or 12 weeks before the index date. Because relatively short time windows were analyzed, an interval period was introduced between the four control periods and the hazard period to minimize the possibility of overlapping prescriptions between these periods. Accordingly, an interval of 12 weeks was selected between the end of the control period and the beginning of the hazard period to avoid a carryover effect. 

### 2.5. Statistical Analysis

Purgative exposure during the hazard period and matched control periods was investigated. A difference of several days or more between the prescription date and actual administration date of purgatives may be present owing to the waiting time for a colonoscopy appointment. Considering the actual clinical situation, the purgative exposure date was defined as the day before the date of the colonoscopy procedure. Purgative use between the control and hazard periods was compared using conditional logistic regression analysis, and the odds ratios (ORs) with 95% confidence intervals (CIs) were estimated. Owing to the case-crossover study design, the outcomes were free from all personal and time-invariant confounders. Statistical analysis was performed using SAS (version 9.4; SAS Institute, Inc., Cary, NC, USA).

## 3. Results

**Figure 2:**
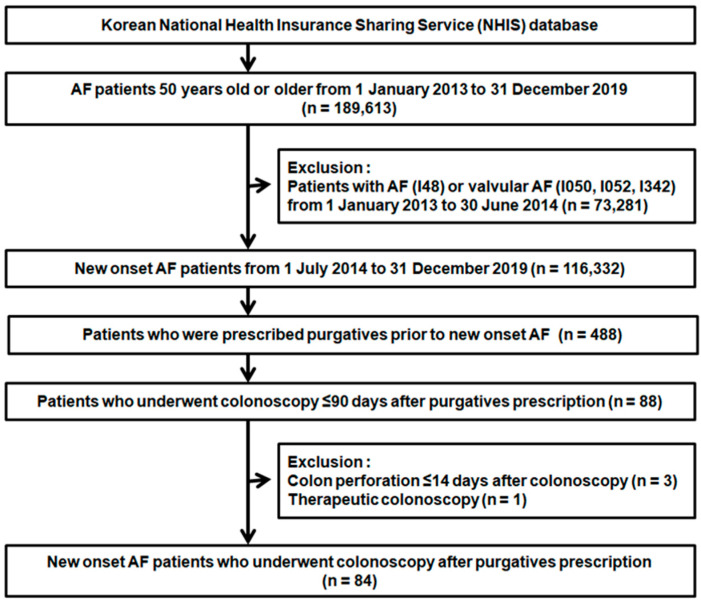
Flow diagram of the study population. AF, atrial fibrillation.

A total of 189,613 patients with AF aged ≥50 years were identified from the NHIS database between 1 January 2013 and 31 December 2019. To include only patients with new-onset AF, 73,281 patients with AF or valvular AF between 1 January 2013 and 30 June 2014, were excluded. Among the remaining 116,332 patients, 488 were prescribed purgatives before they developed new-onset AF. Of these 488 patients, 88 underwent colonoscopy within 90 days after purgative prescription. Three patients who experienced colon perforation after colonoscopy and one patient who underwent a therapeutic colonoscopy procedure, as described above, were excluded. Finally, 84 patients were included in the analysis (Figure 2). 

The baseline characteristics of the study population are summarized in Table 1. 

The mean patient age was 72.4 ± 9.1 years, and the proportion of men was 63.1%. A large proportion of the patients had comorbidities, including hypertension (89.3%), diabetes mellitus (67.9%), ischemic heart disease (54.8%), and heart failure (25.0%). The most frequently prescribed purgative was 2 L PEG-A (*n* = 56, 66.7%), followed by 4 L PEG (*n* = 21, 25.0%). A few patients were prescribed other purgatives, such as OSS (*n* = 4, 4.8%), SPMC (*n* = 2, 2.4%), and SPS (*n* = 1, 1.2%). None of the patients were prescribed NaP.

Table 2 presents the concordant and discordant pairs of purgative exposures observed among the patients with new-onset AF between the control periods and the hazard period according to the specified time windows. 

Because we paired one hazard period and four control periods for each patient, the number of pairs exposed in the hazard periods shown in Table 2 is equal to four times the number of patients exposed to purgatives in the hazard periods presented in Table 3. 

The number of exposures to bowel preparation during the control periods and the hazard period were compared. The association of bowel preparation and subsequent colonoscopy with new-onset AF was stratified according to the time windows in Table 3. The proportion of patients with bowel preparation was higher during the hazard period than during the control periods: 11.9% vs. 4.2%, 13.1% vs. 4.8%, 16.7% vs. 6.3%, 28.6% vs. 11.9%, and 29.8% vs. 14.0% in the 1-, 2-, 4-, 8-, and 12-week time windows, respectively. Regardless of the time window, statistically significant associations were found between bowel preparation exposure and the occurrence of AF. In the 1-week period after bowel preparation exposure, the risk of AF was 3.11-fold higher than that in the control periods (95% CI 1.33–7.27). Similarly, in the 2-, 4-, 8-, and 12-week time windows, the risk of AF was 3.01 (95% CI 1.34–6.77), 3.00 (95% CI 1.45–6.19), 2.96 (95% CI 1.66–5.27), and 2.61 (95% CI 1.49–4.56) times higher than that at the other control times. The risk of AF due to bowel preparation and colonoscopy was the highest during the first (1-week) time window, and as the time window increased, the risk gradually decreased.

With respect to comorbidities, bowel preparation and colonoscopy was significantly associated with the occurrence of AF in all time windows even among patients without heart failure. In addition, a significant association was found in the 8-week time window even among patients without diabetes mellitus and in the 1-, 8-, and 12-week time windows even among patients without ischemic heart disease (Table 4).

## 4. Discussion

In this nationwide population-based case-crossover study, we found that bowel preparation and subsequent colonoscopy was associated with the risk of AF in patients aged ≥50 years. The significant association of bowel preparation and colonoscopy with the risk of AF persisted in all different time windows, and the risk of AF tended to decrease as the time window increased.

As most patients used PEG-based solutions for colonoscopy, our results suggested that choosing PEG-based bowel preparations did not help avoid the risk of AF. In this study, 2 L PEG-A and 4 L PEG were used by 66.7% and 25.0% of the patients, respectively. PEG is the most commonly used agent for colon cleansing because it is a non-absorbable, non-digestible, osmotically balanced laxative lavage solution that rarely affects body fluid and electrolyte balance [19]. Given the already established efficacy and few known adverse events of PEG solutions, PEG-based preparations are the most suitable and safe colon cleansing agents for patients with comorbidities or elderly patients [20]. However, some recent case reports have raised safety concerns related to the occurrence of cardiac events after the use of PEG-based bowel preparations. A few case reports involving patients who experienced heart failure exacerbation after using PEG-based bowel preparations for colonoscopy have been published [4,5,6]. Furthermore, another case report has suggested the possible association of PEG use with the risk of cardiac arrhythmia [21]. Additionally, as mentioned in the Introduction section, a case series report on two patients who developed AF after PEG use has also been published [7]. This case series is the only report worldwide to suggest an association between PEG use and AF. In this case series, the two patients were aged 68 and 69 years. Both patients had substantial atrial dilatation, which might have contributed to the genesis and persistence of AF. Atrial dilatation induces structural and electrical remodeling of the atria and increases the number of atrial problems that can accommodate reentry circuits, which is one of the pathophysiological mechanisms of AF [22]. Most patients in our study had hypertension, and more than half had ischemic heart disease. 

Aging is also an important risk factor for AF, as it triggers variable changes in atrial refractoriness, such as reduced atrial conduction velocity and increased electrogram fractionation [23]. The mean patient age in our study was 72.4 years, and 68% of the patients were aged ≥70 years. Our findings indicate that elderly patients may be more susceptible to cardiac complications associated with the use of purgatives. In addition, most of the included patients had cardiac comorbidities. Because PEG is known to be a relatively safe bowel preparation agent, it tends to be used without much caution. However, on the basis of our results, PEG should be used with caution in patients undergoing colonoscopy, especially in elderly patients with cardiac comorbidities. 

The present study found a significant association of bowel preparation and colonoscopy with the occurrence of AF, even in patients without heart failure and those without ischemic heart disease. Therefore, the association between the two factors cannot be simply explained by an underlying heart disease. Although the mechanisms linking bowel preparation and the risk of AF could not be clearly elucidated, some potential explanations may be proposed. First, bowel preparation can induce electrolyte abnormalities, particularly hypokalemia. Although we were unable to investigate electrolyte levels, electrolyte abnormalities such as hypokalemia might have caused AF in our patients. Hypokalemia alters, and sometimes even depolarizes, the resting potential (Vr). It can also significantly prolong the final repolarization phase of the atrial action potential, leading to myocardial excitability and refractoriness [24]. Additionally, alterations in the intracellular electrolyte balance can modify Vr and atrial action potential repolarization and can cause a change in the atrial electrophysiological substrate [24]. In fact, there is a case report describing a patient who developed hypokalemia causing cardiac arrhythmia after the use of PEG-based bowel preparations for colonoscopy [21].

Second, the combined occurrence of rapid gastrointestinal motility, luminal distension, and colonic secretion during bowel preparation may induce an increase in the parasympathetic tone. Parasympathetic stimulation via the vagus nerve may be a favorable factor contributing to the pathogenesis of AF. Parasympathetic stimulation causes electrophysiological changes in the atrium through acetylcholine released by vagus nerve activation [25]. Acetylcholine activates the cardiac muscarinic receptors, which regulate membrane ion channels through direct activation of potassium channels that accelerate repolarization and induce hyperpolarization [26].

The final factor that can explain the association of bowel preparation and subsequent colonoscopy with AF may be the patients’ anxiety and emotional stress about undergoing colonoscopy. Some studies have reported that >50% of patients have moderate to severe anxiety before colonoscopy [27]. Activation of the sympathetic nervous system due to anxiety and emotional stress may contribute to the development of AF because it increases calcium influx and shortens the duration of atrial action potentials [28]. Moreover, anxiety states can cause catecholamine overload, which can lead to the formation of an arrhythmogenic substrate and can trigger the onset of AF [29]. Additionally, anxiety is linked to systemic inflammation, which can lead to the onset and maintenance of AF [29]. Systemic inflammation plays a pivotal role in the development of AF through atrial fibrosis, irregular myocellular hypertrophy, and myocyte apoptosis or necrosis [29,30]. 

This is the first study worldwide to demonstrate the relationship between the risk of AF and bowel preparation and subsequent colonoscopy. However, our results should be interpreted considering some limitations. First, although AF should be diagnosed using electrocardiography (ECG), we defined incident cases of AF as hospital visits or admissions with a diagnosis of AF in the NHIS database because the database does not contain ECG results. We did not directly evaluate the accuracy of the definition of AF in this study. However, a previous study using the same definition of AF as ours compared the diagnosis derived from the NHIS database with the actual diagnosis based on ECG in the medical records, and their validation analysis showed a very high positive predictive value (94.1%) [18]. Second, as the number of patients with new-onset AF was very small, we could not compare the risk of AF according to the types of purgatives. Third, it was impossible to directly elucidate the pathophysiological mechanism linking bowel preparation/colonoscopy and AF because electrolyte levels and echocardiographic findings for the study participants were not included in the NHIS database. Forth, we could not identify the exact association between bowel preparation itself and the risk of AF because patients that are exposed to bowel preparation are at the same time exposed to colonoscopy in this study. Colonoscopy itself or sedative agents could also influence on the development of AF. Finally, since the majority of patients in our study were older adults and had cardiac comorbidities, our results cannot be extrapolated to the general population. Future studies in younger patients without cardiac comorbidities are needed to verify the association of bowel preparation and colonoscopy with AF.

## 5. Conclusions

In conclusion, bowel preparation and undergoing colonoscopy is associated with the risk of AF. Selecting PEG-based bowel preparations cannot ensure freedom from the risk of AF. Bowel preparation and subsequent colonoscopy needs to be performed with caution particularly in elderly patients with hypertension, regardless of the selected type of purgative agent.

## Figures and Tables

**Table 1:** Characteristics of the study population (*n* = 84).

Variable	Value
Sex, *n* (%)	
Male	53 (63.1)
Female	31 (36.9)
Age, mean ± standard deviation, years	72.4 ± 9.1
Age, years, *n* (%)	
50–59	10 (11.9)
60–69	17 (20.2)
70–79	37 (44.0)
≥80	20 (23.8)
Comorbidities, *n* (%)	
Hypertension (I10–I15)	75 (89.3)
Diabetes mellitus (E10–E14)	57 (67.9)
Ischemic heart disease (I20–I25)	46 (54.8)
Heart failure (I50)	21 (25.0)
Year of cohort entry, *n* (%)	
2014	7 (8.3)
2015	33 (39.3)
2016	25 (29.8)
2017	12 (14.3)
2018	4 (4.8)
2019	3 (3.6)
Types of purgatives, *n* (%)	
PEG 2 L + ascorbic acid	56 (66.7)
PEG 4 L	21 (25.0)
OSS	4 (4.8)
SPMC	2 (2.4)
SPS	1 (1.2)
NaP	0

PEG, polyethylene glycol; OSS, oral sulfate solution; SPMC, sodium picosulfate + magnesium oxide + citric acid; SPS, sodium picosulfate + PEG + D-sorbitol; NaP, sodium phosphate.

**Table 2:** Concordant and discordant pairs of purgative exposures observed among patients with new-onset atrial fibrillation between the hazard and control periods according to time windows.

Time Window	Hazard Period	Control Period	
		Non-Exposed	Exposed
1 week	Non-exposed	292	4
	Exposed	30	10
2 weeks	Non-exposed	287	5
	Exposed	33	11
4 weeks	Non-exposed	273	7
	Exposed	42	14
8 weeks	Non-exposed	224	16
	Exposed	72	24
12 weeks	Non-exposed	214	22
	Exposed	75	25

**Table 3:** Association of bowel preparation and subsequent colonoscopy with new-onset atrial fibrillation according to time windows.

Time Window	Exposed to Purgatives in 84 Hazard Periods, *n* (%)	Exposed to Purgatives in 336 Control Periods, *n* (%)	OR (95% CI)	*p*-Value
1 week	10 (11.9)	14 (4.2)	3.11 (1.33–7.27)	0.009
2 weeks	11 (13.1)	16 (4.8)	3.01 (1.34–6.77)	0.008
4 weeks	14 (16.7)	21 (6.3)	3.00 (1.45–6.19)	0.003
8 weeks	24 (28.6)	40 (11.9)	2.96 (1.66–5.27)	0.001
12 weeks	25 (29.8)	47 (14.0)	2.61 (1.49–4.56)	0.001

OR, odds ratio; CI, confidence interval.

**Table 4:** Association of bowel preparation and subsequent colonoscopy with new-onset atrial fibrillation in patients without comorbidities.

Without Comorbidities	1 Week		2 Weeks		4 Weeks		8 Weeks		12 Weeks	
	OR (95% CI)	*p*-Value	OR (95% CI)	*p*-Value	OR (95% CI)	*p*-Value	OR (95% CI)	*p*-Value	OR (95% CI)	*p*-Value
Without hypertension	4.04 (0.25–65.20)	0.326	2.01 (0.18–22.46)	0.570	2.00 (0.18–22.45)	0.570	3.07 (0.67–14.01)	0.147	2.45 (0.57–10.47)	0.226
Without diabetes mellitus	3.07 (0.67–14.01)	0.147	3.31 (0.87–12.61)	0.079	2.35 (0.67–8.22)	0.181	2.62 (1.05–6.53)	0.040	2.25 (0.92–5.51)	0.075
Without ischemic heart disease	3.48 (1.04–11.70)	0.044	2.97 (0.92–9.62)	0.069	2.51 (0.88–7.11)	0.084	2.66 (1.21–5.88)	0.015	2.26 (1.04–4.89)	0.039
Without heart failure	3.11 (1.21–8.00)	0.019	2.84 (1.12–7.20)	0.028	3.01 (1.34–6.77)	0.008	2.78 (1.46–5.29)	0.002	2.51 (1.35–4.67)	0.004

OR, odds ratio; CI, confidence interval.

## Data Availability

The data presented in this study are available on request from the corresponding author.
